# The energetics of protein–lipid interactions as viewed by molecular simulations

**DOI:** 10.1042/BST20190149

**Published:** 2019-12-24

**Authors:** Robin A. Corey, Phillip J. Stansfeld, Mark S.P. Sansom

**Affiliations:** 1 Department of Biochemistry, University of Oxford, South Parks Road, Oxford OX1 3QU, U.K.; 2 School of Life Sciences and Department of Chemistry, University of Warwick, Coventry CV4 7AL, U.K.

**Keywords:** free energy, lipid, membrane protein, molecular dynamics, simulation

## Abstract

Membranes are formed from a bilayer containing diverse lipid species with which membrane proteins interact. Integral, membrane proteins are embedded in this bilayer, where they interact with lipids from their surroundings, whilst peripheral membrane proteins bind to lipids at the surface of membranes. Lipid interactions can influence the function of membrane proteins, either directly or allosterically. Both experimental (structural) and computational approaches can reveal lipid binding sites on membrane proteins. It is, therefore, important to understand the free energies of these interactions. This affords a more complete view of the engagement of a particular protein with the biological membrane surrounding it. Here, we describe many computational approaches currently in use for this purpose, including recent advances using both free energy and unbiased simulation methods. In particular, we focus on interactions of integral membrane proteins with cholesterol, and with anionic lipids such as phosphatidylinositol 4,5-bis-phosphate and cardiolipin. Peripheral membrane proteins are exemplified via interactions of PH domains with phosphoinositide-containing membranes. We summarise the current state of the field and provide an outlook on likely future directions of investigation.

## Introduction

Biological membranes are formed from a bilayer containing different species of lipid, into which integral transmembrane proteins are inserted, whilst peripheral membrane proteins are bound to the surface. These membrane proteins play key roles in cell function via control of cellular transport, metabolism, and signalling. Many membrane proteins are of biomedical significance, and are therefore drug targets.

## Protein–lipid interactions: structures and simulations

Membrane proteins interact with multiple lipid species. Some interactions play a structural role, e.g. stably anchoring and/or promoting oligomerization an integral protein within a bilayer [1,2], or targeting a peripheral protein to the surface of a specific cellular membrane. Other interactions influence the function of the protein, either allosterically [3–6] or via a direct functional role [7,8]. The nature of these lipid interactions is, therefore, the subject of both experimental and computational analyses [6].

**Figure 1.**
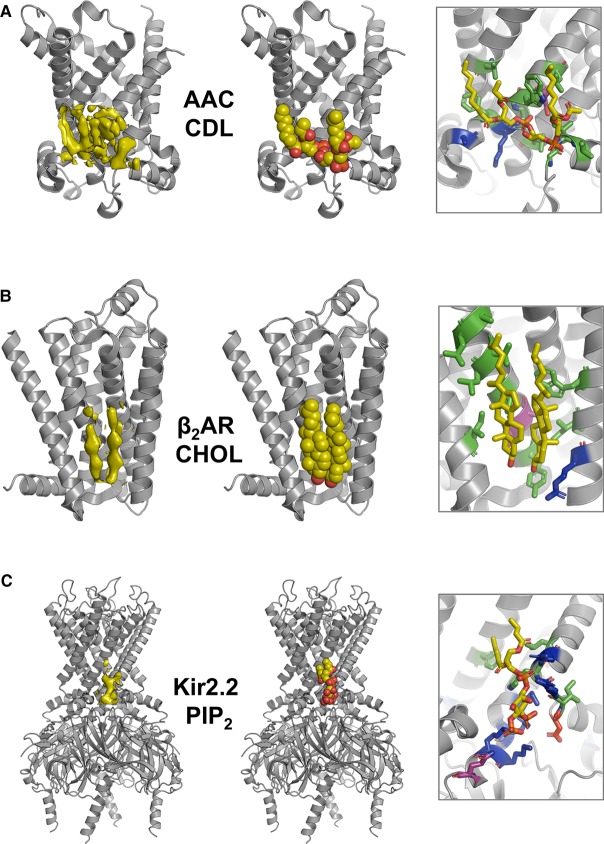
Identification of protein–lipid interactions. (**A)** Left: view of bovine AAC (PDB 1OKC), with the protein shown as a grey cartoon and densities for CDL shown in yellow surface. Middle: A bound CDL molecule from the crystal structure is shown in yellow, orange, and red spheres. Right: close-up of one of the CDL molecules: the CDL and contacting residues are shown as sticks, with aromatic, hydrophobic, and small amino acid sidechains coloured green, acidic sidechains red, basic sidechains blue, and polar uncharged sidechains in purple. (**B**) As (**A**), but for human β_2_AR (PDB 3D4S), showing the binding mode of two cholesterol molecules. (**C**) As (**A**) but for *G. gallus* Kir2.2 (PDB 3SPI) in complex with di-C8-PIP_2_ molecules.

Structural studies, using X-ray crystallography and/or cryo-electron microscopy, often require solubilisation of membrane proteins in detergents, which removes the majority of the bound lipids. Some protein–lipid interactions survive this process, allowing identification of lipids in the resultant density maps (e.g. [9–13]; Figure 1; Table 1). The use of nanodiscs, a portion of lipid bilayer encircled by either protein [14] or amphipathic polymer (SMALP) [15], may aid this purely structural approach (e.g. [16]). However, a combination of modest resolution and/or partial occupancy of the lipid site and the structural similarities between related lipid molecules can make unambiguous assignment of the identity of the bound lipid molecule non-trivial. In addition, it is uncertain to what extent interactions seen in a purified complex reflect those present in the native membrane environment.

**Table 1:** Where reported, errors are included for the free energy (FE) estimate. Note that the method of how the error is calculated varies between different studies

Lipid	Protein	PDBs	FE (kJ mol^−1^)	Ref.
Chol.	Smoothened	5L7D	−10	[67]
	β2 AR inactive	3D4S	−14.3, −14.6	[65]
	β2 AR active	3SN6	−9.8, −13.3	[65]
	A_2A_R inactive	4EIY	−9.4, −9.6, −11.7	[65]
	A_2A_R active	3QAK	−14.4, −10.6, −9.9, −9.3	[65]
	β2 AR	3D4S	−53 ± 0.8	[66]
	µ-opioid	5C1M	−12 ± 3.8	[66]
	5-HT_2B_	4NC3	−18 ± 2.1	[66]
	A_2A_R	5IU4	−5 ± 1 to −9 ± 3	[51]
	A_2A_R-adenosine	2YDO	−3, −5	[64]
	PgP	Model	−26 ± 3, −6 ± 1, −4 ± 1	[68]
	Kir2.1	Model	−3.3 ± 6, −7.5 ± 8, −10.5 ± 6	[69]
	PC2	6T9N	−12 ± 3	[32]
CDL	AAC	1OKC	−20 ± 5 to −25 ± 5	[29]
	AAC	1OKC	−22	[47]
	AAC	1OKC	−5 ± 2 to −14 ± 3	[51]
	LeuT	2A65	−6 ± 3 to −9 ± 2	[51]
	Cytochrome c oxidase	1OCC/2OCC	−10 to −35	[24,78]
PIP_2_	Kir2.2 (truncated)	3SPI	−42 ± 5	[47]
	Kir2.2 (full)	3SPI	−45 ± 6 to −48 ± 2	[51]
	PC2	6T9N	−37 ± 3	[32]
	A_2A_R inactive	3EML	−10 to −20	[44]
	A_2A_R active	5G53	−10 to −50	[44]
	A_2A_R active + mini Gs	5G53	−20 to −80	[44]
	EGFR transmembrane	2M20	−42 ± 5	[75]

Note that the method of how the error is calculated varies between different studies.

Computational approaches [17], especially molecular dynamics (MD) simulations [18], provide a powerful tool for analysis of protein–lipid interactions. Recent improvements in lipid force fields have greatly improved research in this area, however, these lipid force fields still have limitations which must be taken into consideration when analysing data (for recent discussions, see [19–21]). Parallel advances in MD algorithms and computer hardware now permit analysis of interactions that occur on relatively long (microsecond) timescales. For example, in certain coarse-grained (CG) biomolecular force fields, such as Martini [22], groups of atoms are modelled as a single bead or particle, allowing faster sampling of protein–lipid configurations compared with atomistic simulations. CG simulations have been successfully applied to predicting the location of a lipid binding site to a given membrane protein for several different systems (e.g. [23–26]; Table 1). These predictions have been validated by comparison with experimental structural and/or biophysical data for many cases, including e.g. phosphatidylinositol (4,5) bis-phosphate (PIP_2_) bound to Kir channels [27,28]; cardiolipin (CDL) bound to the ADP/ATP carrier [29,30], and to the LeuT transporter [1]; and PIP_2_ bound to Class A GPCRS [31] and to TRP channels [32]. MD simulations can also provide information on local enrichment of various lipid species in the bilayer around a given membrane protein [33].

## Computational estimation of lipid-binding energetics

MD simulations have been demonstrated to predict binding sites, allowing structural aspects of protein–lipid interactions to be analysed (Figure 1 and Table 1). However, it is also important to understand the affinities of these interactions. The crowded nature of a cell membrane environment [34–36] means that many different species will jostle for position around a membrane protein, with many lipids potentially able to bind. Knowledge of the interaction free energies of different lipid species for a specific site on a protein will help to indicate how likely such an interaction is to occur within a native cell membrane, rather than e.g. in a detergent-solubilised experimental environment.

Importantly, lipids are not only potential ligands for a membrane protein, they also act as the surrounding environment. Thus, all lipid binding sites on the transmembrane surface of a protein are likely to be occupied by one or another lipid species. Therefore, it is important to be able to estimate the free energy of binding for a specific lipid to a given site compared with that of the other major lipid species in the surrounding bilayer.

**Figure 2.**
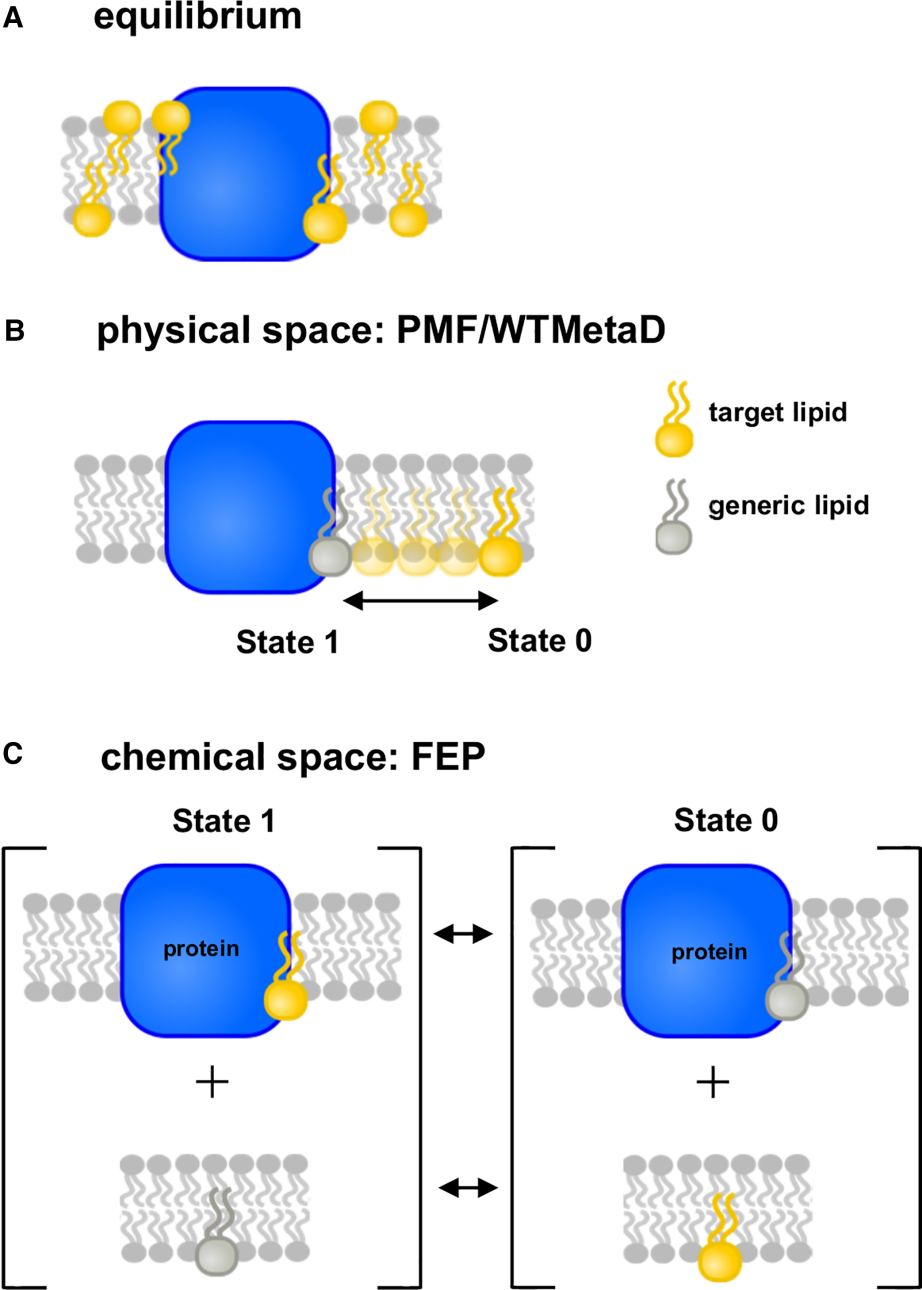
Equilibrium and free energy calculations for protein–lipid interactions. (**A**) Equilibrium simulations allow multiple protein–lipid events to occur in an unbiased fashion over long MD simulations. (**B**) Construction of a reaction co-ordinate in physical space requires sampling of the protein–lipid complex (State 1) and of the lipid free in membrane (State 0) as well as many intermediate positions of the lipid between the binding site and surrounding bilayer bulk. Note that in State 0, the lipid binding site on the protein will be occupied by a generic lipid. (**C**) Sampling States 1 and 0 in chemical space. This is done over two sets of simulations: first, whilst bound to the protein, the target lipid (yellow) is alchemically transformed into a background (generic e.g. PC; grey) lipid (upper panels). At the same time, a background lipid free in the bilayer is also transformed in a target lipid molecule. Combining the upper and lower calculations yields the free energy for binding of the target lipid relative to that of a background lipid.

Computational approaches to study the free energies of protein–lipid interactions can be broadly divided into two categories: those using long, unbiased MD simulations to estimate the probability (and hence free energy) of interactions directly (Figure 2A), and those which use biased (i.e. more targeted) MD simulations to compute the free energy of interaction of a specific lipid molecule with a protein.

Both approaches aim to compute the free energy difference between two states of a system: State 1 (Figure 2B) corresponds to a specific ‘target’ lipid bound to the protein, and State 0 to the target lipid free in the surrounding membrane. In State 0, a different (bulk or background) lipid species from the surrounding bilayer environment is likely to interact with the protein. Therefore, the quantity of interest is the ΔΔ*G* of binding, where:

$$\Delta \Delta G=\Delta G_{\mathrm{target}}-\Delta G_{\mathrm{bulk}}$$

This is analogous to a binding site on a (water soluble) protein which can be occupied by either a ligand in the holo state or water molecules in the apo state.

### Equilibrium simulation studies

Conceptually, the simplest approach to estimating free energies of protein–lipid binding is via long, unbiased simulations. These have the advantage that they are relatively straightforward to perform and analyse [37–40]. However, depending on the underlying free energy landscape they may require extended simulation times to adequately sample possible configurations of interest.

In principle, equilibrium simulations provide estimates of the relative probabilities of the lipid being bound (*P*_bound_) or free (*P*_free_) (where *P*_bound_ *= *1 − *P*_free_), calculated from the observed fraction of time a simulation system spends in the bound (State 1) and unbound (State 0) states. These can then be used to calculate the ΔΔ*G* of the protein–lipid interaction:

$$\Delta \Delta G=\;-RT\ln\left(\frac{P_{\mathrm{bound}}}{P_{\mathrm{free}}}\right)$$

It should be noted that for these probabilities to be properly sampled, multiple binding, and dissociation events must be observed, and the simulation time for this is difficult to be estimate in advance as it will depend on the specific protein–lipid interaction being modelled, and plausibly testing the limits of current MD simulations. If a sufficient number of binding and dissociation events occur, it may be possible to estimate *k*_on_ and *k*_off_ either directly from the simulation trajectories, possibly aided by e.g. Markov state models [41] or MetaDynamics [42]. Indeed, the latter approach has recently been applied to the calculation *k*_off_ for ligand dissociation from a (water soluble) protein [43].

Equilibrium simulations also have the advantage that mixed lipid bilayers can readily be evaluated. In these cases, ΔΔ*G* refers to the free energy of interaction of a target lipid in relation to the other lipid species in the system. This is, in turn, closer to a complex cell membrane than a free energy simulation based on simple single species lipid bilayer environment. In addition, multiple binding sites can be analysed simultaneously (Figure 2A).

### Potential of mean force calculations

There are many more ‘targeted’ approaches to estimating free energies of protein–lipid interactions, of which potential of mean force (PMF) calculations are the most widely used (e.g. [24,29,44]). These can provide a cross-section through a more complex free energy landscape, showing the free energy of a lipid/protein system as a function of a typically one-dimensional reaction co-ordinate, e.g. the distance between the headgroup of a specific lipid molecule and its binding site on a membrane protein (i.e. the distance between States 1 and 0 in Figure 2B). Many methods are available (e.g. umbrella sampling [45] or replica exchange umbrella sampling [46,47]) to estimate the free energy as a function of the reaction co-ordinate.

To speed up convergence, it is typical to perform simulations with one copy of the target lipid, and the rest of the membrane modelled with a single generic lipid e.g. phosphatidylcholine (PC), rather than a complex lipid mixture. Therefore, comparisons of Δ*G*s for the target lipid with a generic lipid (often close to 0 kJ mol^−1^) allows estimation of ΔΔ*G* between the two lipid species.

### Free energy perturbation and absolute binding free energy calculations

More recently, studies have attempted to directly calculate ΔΔ*G* using either free energy perturbation (FEP) [48] or absolute binding free energy (ABFE) [49] calculations. For these, the target lipid molecule is ‘alchemically’ transformed *in silico* into a generic lipid (see [50]), or fully removed from the system. If this is done to a target lipid whilst in complex with a protein and again to the same lipid species free in the bilayer, the difference between these values yields the free energy associated with the lipid binding to the protein. This permits modelling of the same states as by using PMF, but through a co-ordinate in chemical space (Figure 2C), rather than physical distance. Recent work suggests that this gives equivalent values to PMF analyses, while using less computational resource [51]. However, both approaches provide information on a single lipid site only, which needs to be identified prior to running calculations.

### Metadynamics

MetaDynamics allows more extensive sampling of complex free energy landscapes. For example, in well-tempered metadynamics (WTMetaD) [52], the sampling of a free energy landscape is enhanced in a history-dependent manner [53] to ensure that the simulation system spends less time in energy minima, thus significantly speeding up the overall process. If applied to a patch of the membrane around a protein of interest, with a collective variable (CV) corresponding to the distance between the lipid and protein in the bilayer plane, this can be used to compute the binding poses and free energies for multiple lipid binding sites around a protein. In test calculations, this approach revealed all four PIP_2_ binding sites on Kir2.2 and all three CDL sites on AAC (see below), and yielded free energies of interaction in good agreement with both PMF and FEP/ABFE [51], albeit at a greater computational cost.

## Application to specific lipids

Computational studies of protein–lipid interactions have largely focussed on two main classes of lipid: cholesterol, which as a relatively hydrophobic and rigid lipid species is amenable to free energy calculations, and more complex anionic lipids e.g. CDL and PIP_2_, which interact with a range of membrane proteins in bacterial and mitochondrial inner membranes [54], and in eukaryotic cell membranes [55,56], respectively.

### Binding of cholesterol to GPCRs and other membrane proteins

Cholesterol is present in high (>30%) concentrations in eukaryotic plasma membranes [57]. Cholesterol interacts with many different proteins, including G-protein coupled receptors (GPCRs) and ion channels [6]. GPCRs constitute one of the largest classes of membrane proteins, and their key roles in many biological processes mean that they make up approximately one-third of all drug targets [58]. Many GPCRs have been shown to bind [59,60] and have been suggested to be functionally modulated by cholesterol [61–63]. These include the intensively studied adenosine 2A (A_2A_R) and β2-adrenergic (β-2AR) receptors.

**Figure 3.**
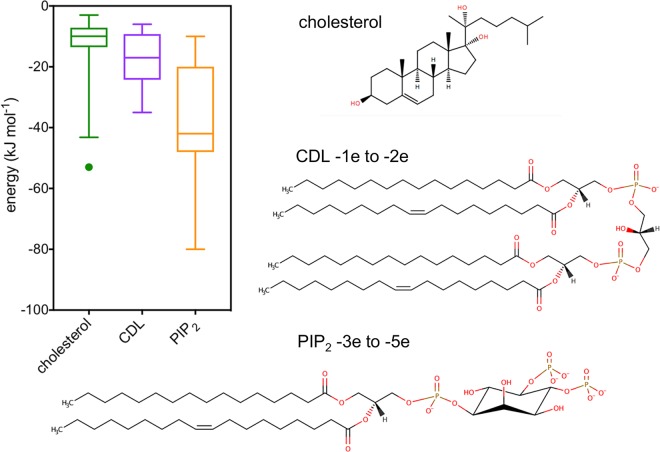
Computational estimates of protein–lipid interaction free energies. (**A**) Summary of estimates of free energies for integral protein interactions of cholesterol (green), CDL (purple), and PIP_2_ (orange). The data for this graph were derived from the range of simulation studies (both coarse-grained and atomistic) discussed in the main text. Data are plotted as box and whiskers, showing the 5–95 percentile range. Data plotted in Prism 7 (GraphPad). (**B**) Cholesterol, CDL, and PIP_2_, indicating the likely charge state of the anionic lipids.

The free energies of cholesterol–GPCR interactions have been addressed in many simulation studies (Figure 3). Cholesterol interactions with A_2A_R have been estimated by both WTMetaD and ABFE calculations using the Martini CG force field [51], yielding values from ∼ −5 to −10 kJ mol^−1^. Cholesterol–GPCR interactions have also been modelled using unbiased equilibrium simulations. Sub-microsecond duration atomistic simulations of A_2A_R in a 30% cholesterol and 70% PC membrane [64] yielded free energies of interaction between −3 and −5 kJ mol^−1^ for different sites, in good agreement with the values from the Martini CG estimates above. Extended (50 µs) CG simulations in membranes containing 15% cholesterol [65] yielded interaction free energies of −9 to −12 kJ mol^−1^ for the agonist-free state of the A_2A_R. Thus, for class A GPCRs a consensus emerges (despite using different estimation methods and force fields) of free energies of interaction in the range −5 to −12 kJ mol^−1^.

Other free energy calculation approaches have also been used to estimate the strength of cholesterol interactions. Salari et al. [66] used ABFE with the atomistic CHARMM36 force field to obtain values of cholesterol binding to β-2AR that is much stronger than in other studies (∼ −53 kJ mol^−1^). Interestingly, they obtained free energies for other different GPCRs, µ-opioid (∼ −12 kJ mol^−1^) and 5-HT_2B_ (∼ −18 kJ mol^−1^) more in line with the energies obtained from other calculations.

Comparisons with proteins other than Class A GPCRs also suggest that cholesterol binds with modest (i.e. ∼ −10 kJ mol^−1^) free energies to surface-exposed sites on TM domains. CG calculations yielded free energies of −10 kJ mol^−1^ for cholesterol interactions with the TM domain of the Class F GPCR Smoothened [67]. Comparable values (−4 to −6 kJ mol^−1^) were obtained by CG simulations for two different cholesterol binding sites on the ABC transporter P-glycoprotein transporter [68]. Interestingly, this study also revealed a third, more deeply buried, site with a stronger interaction of −25 kJ mol^−1^. Similarly, atomistic simulations employing molecular mechanics/Poisson–Boltzmann surface area (MM-PBSA) calculations suggest binding energies of −3, −7.5, and −10.5 kJ mol^−1^ of cholesterol to three distinct sites on Kir2.1 [69]. Recent CG simulations based on cholesterol bound to the ciliary TRP channel PC2 [32] yielded an interaction free energy of −12 kJ mol^−1^. Thus, these systems also reveal relatively modest binding free energies for cholesterol. This is also in agreement with a recent docking study of cholesterol to a range of different GPCRs which concluded that cholesterol does not tend to occupy a single, well-defined conformational state but when bound to a TM surface site can adopt a range of binding poses [70], consistent with a modest free energy of interaction (Figure 3).

Experimental data against which to compare these calculations are relatively sparse. For example, it has been suggested for the β_2_AR that low affinity (∼ −10 kJ mol^−1^) and high affinity (∼ −50 kJ mol^−1^) cholesterol sites may exist, by NMR and by thermostability assays, respectively [71]. However, the relationship of these binding modes to the computational analyses described above (and to the 3–5 cholesterol binding sites on the β_2_AR indicated by structural studies) remains to be established.

### Binding of anionic lipids to membrane proteins

Many membrane proteins interact specifically with anionic lipids, including e.g. CDL and PIP_2_. PIP_2_ is a signalling lipid found in the eukaryotic cell membrane and has been shown to with a wide range of receptors [6], ion channels [56], and transport proteins [72]. These interactions have been the subject of range of simulation studies [18].

Inward rectifying potassium channels (Kirs) provide a well-studied example of activation of an ion channel by PIP_2_ binding [11]. The energetics of these interactions has been explored using PMF calculations, giving free energies of −42 kJ mol^−1^ (for Kir2.2 truncated at the transmembrane region) [47] and of −45 kJ mol^−1^ (for full length Kir2.2) [51]. The free energy of interaction of PIP_2_ with the full length channel has also been estimated by FEP (−48 kJ mol^−1^) and by WTMetaD (−45 kJ mol^−1^) [51]. This can be compared with e.g. the free energy of interaction of PIP_2_ with the transmembrane domain of a TRP channel (PC2) of ∼ −35 kJ mol^−1^ [32]. As can be seen in Figure 1C, the PIP_2_ binding site is formed of a cluster of basic sidechains located close to the interface between the membrane and cytosolic regions. These values of the free energy of interaction may be compared with an experimental estimate of −33 kJ mol^−1^ derived from an EC_50_ for activation of Kir.2 1 by di-C8-PIP_2_ [73].

PIP_2_ interactions have also been investigated with many receptors including GPCRs [31]. For example, free energies for binding to the A_2A_R receptor range from −10 to −80 kJ mol^−1^ depending on the conformational state of the receptor [44]. This state dependence of binding to the receptor suggests PIP_2_ may be an allosteric regulator of A_2A_R, as has been suggested experimentally to be the case for other anionic lipids and GPCRs [74]. PIP_2_ binding free energy has also been estimated at −42 kJ mol^−1^ for a model of the TM domain of the EGFR (a receptor tyrosine kinase) [75]. Together, these data suggest that PIP_2_ binds tightly to membrane proteins at cationic sites at the inner leaflet/cytoplasmic interface systems. In support of this, lengthy simulations suggest that PIP_2_ remains bound for 10 s of microsecond (unpublished data), making a direct sampling of multiple binding/dissociation events computationally challenging.

CDL is an important anionic lipid present in bacterial and inner mitochondrial membranes. CDL binds to many different proteins including the ADP/ATP carrier protein, AAC (also known as the adenine nucleotide translocator, ANT) [9,76,77]. AAC transports ADP and ATP across the mitochondrial inner membrane, allowing regulation of mitochondrial adenine nucleotide concentration. AAC binds and is activated by CDL [9,77]. This interaction has been probed using PMFs, giving a values of the order of −20 kJ mol^−1^ depending on which of the three quasi-equivalent binding sites is being considered [29,47]. Comparable strengths of interaction of CDL with other proteins as estimated using PMF calculations include binding to cytochrome c oxidase (∼ −10 to −35 kJ mol^−1^) [24,78] and also to LeuT, a bacterial inner membrane transporter protein (∼ −6 kJ mol^−1^) [51].

Equilibrium simulations have been used to estimate mean residence times of bound CDL molecules for the mitochondrial F-ATPase [25] (residence times of ∼ 0.5 µs), for AAC (∼ 1–2 µs) [30], for cytochrome c oxidase (∼ 50–60 µs) [24] and for the bacterial SecYEG translocon (∼ 1–2 µs) [26]. In combination with e.g. analysis using Markov state models [41] such simulations could in principle be used to estimate on and off rates for lipids and hence binding affinities.

General trends can be identified from the summary of calculated free energies in Figure 3. As might be anticipated, there is an overall dependence of the strength of protein–lipid interactions on the net charge on the lipid headgroup. However, the range of values for a given lipid species is large (e.g. not all cholesterol sites are the same — see Table 1). Also, for some systems (e.g. PC2 [32]) PIP_2_ is predicted to bind more strongly than PIP_3_ (this is also seen experimentally for e.g. A_2A_R [31]). So although there is an overall dependence on net charge, this is not the sole determinant of the strength of the interaction.

## Membrane surface recognition: PH domains

Membrane signalling and trafficking in eukaryotic cells is regulated by many peripheral membrane protein domains which associate with membrane surfaces, in a lipid-dependent fashion [55]. Considerations of space preclude a more general survey (see e.g. [79–83] for this), so we will focus on one particular example, that of PH (pleckstrin homology) domains. PH domains are a large and well-characterized family present in many membrane recognition proteins, which can bind to phosphatidylinositol-phosphates (mainly PIP_2_ and/or PIP_3_) in membranes in a specific fashion [84]. Cooperativity in binding with other anionic lipid species may aid the recruitment of PH domains to particular cell membranes [85].

Simulation studies have provided a detailed picture of structural and dynamic aspects of the interaction of PH domains with PIP-containing lipid bilayers [86–92]. These studies demonstrate that simulations can predict the interactions of PH domains with lipid bilayers, reproducing the experimental structural aspects seen in interactions of these domains with soluble inositol phosphates which correspond to the headgroups of PIP lipid molecules.

**Figure 4.**
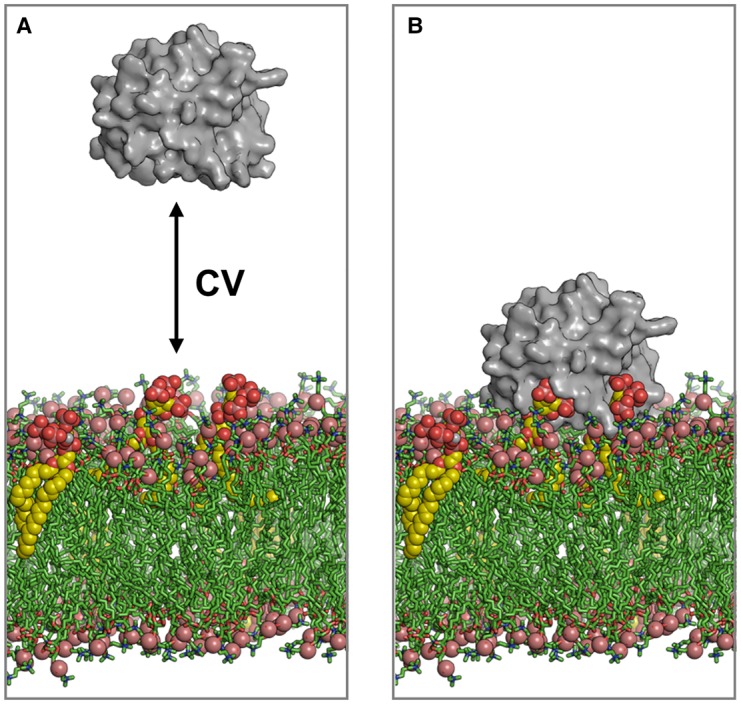
Energetics of interaction of the GRP1 PH domain with a PIP_3_-containing lipid bilayer. (**A**) Simulations of the interaction of the GRP1 PH domain (grey) with a lipid bilayer. The double-headed arrow indicates the reaction co-ordinate for estimation of the free energy landscape (PMF) as a function of the distance of the PH domain from the target lipid molecule(s). (**B**) The GRP1 PH domain is shown bound to a PIP_3_-containing bilayer. PIP_3_ molecules are shown as yellow spheres. Lipids are shown as green sticks. Simulation co-ordinates from [95].

More recently, CG simulations have been used to estimate the free energy of interactions of PH domains with PIP molecules in lipid bilayers. For example, CG simulation studies of the GRP1 PH domain with a PIP_3_-containing lipid bilayer (Figure 4, [93]), with the data suggesting that both canonical and alternative modes of interaction are possible. This was subsequently extended to 12 different PH domains [94] for which structural and energetic experimental data were available. Free energies of the interaction of PH domains with a single PIP molecule in a bilayer ranged from −5 to −40 kJ mol^−1^. Surprisingly, these calculated free energies were generally smaller than those estimated experimentally. Recent simulations [95] suggest that this apparent discrepancy reflects the interaction of a PH domain with multiple PIP molecules when binding to a PIP-containing bilayer. A comparison of the dependence of PH-membrane binding energies on the number of PIP molecules interacting with the PH domain suggests that local nanoscale clustering of PIP molecules can control the strength of this interaction. Thus, binding of a single PH domain to 3 or more PIP molecules yields interaction free energies consistent with experimental estimates of binding affinity of the GRP1 PH domain. Simulations of the PH domain of ACAP1^BAR-PH^ protein [96] and of the PH domain of Bruton's tyrosine kinase [92] have also suggested more than a single PIP binding site.

These examples show how simulations can contribute to our quantitative understanding of membrane recognition by lipid-binding domains. It will be important to extend such studies to other common membrane recognition domains such as C2 domains [83]. Future challenges include extending free energy calculations to include the interactions of more complex multi-domain membrane binding proteins such as the PIP-phosphatase PTEN [97], the dimeric PIP kinase PIP5K1A [98], and the Arf/GEF complex [99].

## Outlook

It is evident that computational methods for studying the energetics of protein–lipid interactions are now established, and are poised to be extended to a wider range of membrane proteins and lipid species in order to address a range of key functional questions. In particular, as increasing computational power allows the description of systems at higher accuracies, such as including polarisability using mean-field corrections [100] or polarisable force fields [101], these analyses are likely to improve in scope and accuracy. It is also evident that we need both more and better experimental data against which to evaluate and compare predictions from simulations. Data are needed both from *in vitro* experiments (ideally in model membranes rather than in detergents) [102–104] and from *in vivo* studies, e.g. single-molecule experiments in live cell membranes [105]. A comparison of such data with simulations will provide a more quantitative understanding of specific protein–lipid interactions in a complex (both compositionally and laterally inhomogeneous) membrane environment. Ongoing improvements in simulation methods and computational power should allow us to extended CG simulations of free energies of interaction to atomistic (but converged!) descriptions. This will require us to account for both dynamic ionisation states of anionic lipids and the binding of divalent metal ions [106–108]. Together, this approach will lead to mechanistic insights into lipids as allosteric ligands, insights which will enable lipid sites to be more fully exploited as druggable sites on biomedically important membrane protein targets.

## Perspectives

- Interactions with specific lipids influence the structure and function of membrane proteins. Identification and characterisation of these interactions is important for our understanding of the biology of these systems, and in the development of novel therapeutics.
- Assessment of the free energies of protein–lipid interactions is central to determining whether the binding is physiologically relevant, especially within the complex environment of the biological membrane. Evaluation of protein–lipid interactions is possible using MD simulations, either through free energy calculations or lengthy unbiased simulations.
- As computer power increases and simulation algorithms improve, so too will the scope and accuracy of these analyses.

## Abbreviations

MD: molecular dynamics
CG: coarse-grained
PIP_2_: phosphatidylinositol (4,5) bis-phosphate
CDL: cardiolipin
PMF: potential of mean force
PC: phosphatidylcholine
FEP: free energy perturbation
WTMetaD: well-tempered metadynamics
CV: collective variable
GPCRs: G-protein coupled receptors
A_2A_R: adenosine 2A receptor
β-2AR: β2-adrenergic receptor
MM-PBSA: molecular mechanics/Poisson–Boltzmann surface area
